# Elucidating the toxicity mechanism of AFM_2_ and the protective role of quercetin in albino mice

**DOI:** 10.1038/s41598-023-28546-8

**Published:** 2023-01-23

**Authors:** Muhammed Onur, Emine Yalçın, Kültiğin Çavuşoğlu, Ali Acar

**Affiliations:** 1 Department of Biology, Institute of Science, Giresun University, Giresun, Turkey; 2 Department of Biology, Faculty of Science and Art, Giresun University, Giresun, Turkey; 3 Department of Medical Services and Techniques, Vocational School of Health Services, Giresun University, Giresun, Turkey

**Keywords:** Biochemistry, Genetics

## Abstract

Aflatoxin M_2_ (AFM_2_) is a type of mycotoxin detected in milk or dairy products from animals consuming contaminated feed. In this study, the toxicity mechanism of AFM_2_ and the protective effects of quercetin were investigated in albino mice. For this purpose, the mice were divided into 6 groups and the groups were fed with quercetin and AFM_2_. The toxic effects of AFM_2_ and the protective properties of quercetin were investigated using physiological, biochemical and cytogenetic parameters. The genotoxic mechanism of AFM_2_ and the protective role of quercetin were investigated by molecular docking, which is an in silico model. As a result, 16 mg/kg b.w AFM_2_ administration caused serious changes in body weight, organ index, kidney and liver weight, and deterioration of antioxidant/oxidant balance in liver and kidney organs. The decrease in glutathione levels along with an increase in malondialdehyde (MDA) levels in the liver and kidney after AFM_2_ administration indicates that oxidative stress is induced. The increases in alanine transaminase (ALT) and aspartat transaminase (AST) levels, which are indicators of liver damage, and the increases in serum levels of blood urea nitrogen (BUN) and creatinine, which are indicators of kidney damage, confirm the damage in both organs. AFM_2_ also caused genotoxicity by inducing micronucleus (MN) and chromosomal abnormalities (CAs) in bone marrow tissue. It has been determined that AFM_2_, which exhibits genotoxicity as a result of its clastogenic and aneugenic effects, causes CAs by interacting with DNA. Quercetin provided significant protection by improving liver and kidney tissues, partial normalization in serum parameter levels, and severe reductions in MN and CAs. The highest protection was determined as 74.1% against dicentric chromosome formations in 50 mg/kg b.w quercetin application. The interaction of quercetin with xanthine oxidase and nitric oxide synthase enzymes was determined in silico with an inhibition constant in the range of 283.71–476.17 nM. These interactions cause changes in the activity of enzymes, reducing the oxidative load in the cell, and in this way, quercetin provides protection. All toxic effects induced by AFM_2_ were decreased with quercetin administration dose-dependently, and this protective effect was associated with quercetin's reduction of oxidative load by inhibiting the free radical-producing enzyme. All toxic effects caused by AFM_2_ were decreased with quercetin administration in a dose-dependent manner, and this protective effect was associated with quercetin's reduction of oxidative load by inhibiting the enzyme that produces free radicals.

## Introduction

Contamination of food products, especially cereals, with aflatoxins produced by *Aspergillus* species raises serious concerns. Aflatoxins are highly toxic compounds and cause serious toxic effects in human and animal tissues by changing metabolic processes. Aflatoxins, which are produced by species such as *Aspergillus flavus* and *Aspergillus parasiticus* and cause adverse effects on food quality, are also associated with various life-threatening diseases in humans and animals. In addition, aflatoxin contamination of nutritious products such as meat and milk obtained from animal foods can cause adverse effects on the health of consumers^1,2^. Aflatoxin B_1_ (AFB_1_), aflatoxin B_2_ (AFB_2_), aflatoxin G_1_ (AFG_1_), aflatoxin G_2_ (AFG_2_), aflatoxin M_1_ (AFM_1_) and aflatoxin M_2_ (AFM_2_) are the major aflatoxin species. Aflatoxin M species, which are hydroxylated metabolites of Aflatoxin B species, pass into milk from the mammary glands of both humans and animals. Approximately 0.3–6.2% of AFB_1_ is transformed into AFM_1_ and passed into milk depending on factors such as the genetics of animals, seasonal variation, milking process and environmental conditions. The presence of aflatoxin M types in milk and dairy products makes it risky to consume these products regularly in daily diets. Especially in products such as cheese and yogurt, aflatoxin M contamination creates serious health problems for consumers, especially liver and kidney diseases^3,4^.

The presence of AFM_1_ and AFM_2_ is detected in animals fed with AFB_1_ and AFB_2_ contaminated feeds. In a study performed in rats, the presence of AFM was detected in the liver and systemic blood after AFB administration. In the same analyzes, no AFB structure was found in the samples in which the presence of AFM was detected, and this was explained by the conversion of all AFB to AFM by biotransformation. AFM types formed in the liver reach the mammary glands and are secreted into the milk^5^. As a result, milk and dairy products obtained from this milk are contaminated with AFM species and reach other organisms. AFM_1_ and AFM_2_, symbolized by "M" because they are milk-derived toxins, are formed as a result of biotransformation of AFB_1_ and AFB_2_, respectively. AFM_2_ is an important metabolite of AFB_2_, and AFB_2_ shows its main effect after it is transformed into AFM_2_ by biotransformation. AFM_2_ is formed as a result of the the biotransformation and hydroxylation of the fourth carbon in the AFB_2_ molecule catalyzed by monooxygenases and passes into the milk. Especially, high milk consumption of children in the developmental age brings to the fore the investigation of the toxicity profile of aflatoxin M species. AFM_2_, with the chemical formula C_17_H_14_O_7_, has a molecular weight of 330.29 g/mol^3,4^. The inadequacy of studies on M_1_ and M_2_ species causes insufficient information on toxic effects. Studies with AFM species in the literature are mostly in the direction of detecting their presence in milk. Considering that contaminated milk and dairy products reach all organisms through nutrition, studies investigating the toxic effects of AFM_1_ and AFM_2_ are very important. In this study, the toxic effects of AFM_2_, for which there is not enough information about its toxicity among aflatoxin species, were investigated. Toxic effects were investigated using different parameters and a versatile toxicity profile was created. The effects of AFM_2_ were investigated physiologically by examining body weight, organ weight and organ index, and biochemically by examining serum parameters, malondialdehyde (MDA) and glutathione (GSH) levels. Micronucleus (MN) and chromosomal abnormality (CA) tests were performed to detect genotoxic effects, and molecular docking, which is an in silico technique, was used to elucidate the toxicity mechanism. In silico techniques are widely used in the safety evaluation of chemicals, elucidating the mechanism and drug interactions. In silico methods also provide very detailed information in the evaluation of toxicological effects^6^. In this study, the data obtained from the in vivo experimental stages were supported by molecular docking, and new findings were provided to the literature regarding the mechanism of genotoxicity of AFM_2_.

The toxic effects induced by many chemicals such as aflatoxins can be reduced by antioxidants consumed in the daily diet. Within the scope of the study, the protective properties of quercetin (3,3',4',5,7-pentahydroxyflavone), which is a powerful antioxidant, against AFM_2_ toxicity were investigated. It is known that quercetin is a powerful antioxidant and in many in vivo studies, it has a protective role against toxic effects by reducing oxidative stress. Quercetin is a plant-derived secondary metabolite belonging to the class of flavonoids. Quercetin, which is found in the seeds, flowers, bark, nuts and leaves of many plants, constitutes approximately 75% of the total flavonoid intake in the daily diet. Quercetin, which has anti-inflammatory, cardioprotective, antioxidant, anti-obesity, anti-mutagen, anti-diabetic, anti-hypercholesterolemic and anti-apoptotic activities, also draws attention with its anti-cancer properties^7,8^. There are many data on the protective properties of quercetin in the literature, but no study has been reported on its effect against AFM_2_ toxicity. In this study, the toxic effects of AFM_2_ and the protective role of quercetin against these toxic effects were investigated in albino mice. Within the scope of the study, AFM_2_-DNA interaction was examined using molecular docking and the mechanism of genotoxicity was clarified. The interaction of quercetin with free radical-producing enzymes was also investigated by molecular docking and the protective effect was associated with the inhibition of these enzymes.

## Materials and methods

Quercetin (250 mg) and all other chemicals were obtained from Sigma-Aldrich and Merck. ALT, AST, BUN and creatinine kits were obtained from Teco Diagnostics (CA).

### Experimental groups

In this study, 36 healthy male *Mus musculus* var. *albinos* were used to determine AFM_2_ toxicity and the protective role of quercetin. During the experiment, albino mice were kept in stainless steel cages at 22 ± 3 °C and 55 ± 5% relative humidity, on a 12-h light/12-h dark cycle. Mice were randomly divided into 6 groups, with 6 mice in each group, and the groups are given in Table 1. It has been reported in many studies in the literature that quercetin provides significant protection against oxidative stress induced by various agents in the range of 10–50 mg/kg b.w^9–11^. In this study, the dose range of quercetin was determined as 25 mg/kg b.w and 50 mg/kg b.w, which is within the range of doses reported in the literature and where the protective role was observed. Quercetin was dissolved in DMSO and the final concentration of DMSO was not more than 0.8%. The preferred dose of AFM_2_ in the study (considering its water solubility of 2.16 g/L) was determined as 16 mg/kg b.w, which is the dose at which AFM_1_, an aflatoxin metabolite with a similar chemical structure to AFM_2_, exhibits toxicity^5^. All experiments were performed in accordance with the guidelines of the Animal Experiments Local Ethics Committee of Giresun University and approved by the Animal Ethics Committee of Giresun University (protocol number: 2017/02). This study was carried out in compliance with the ARRIVE guidelines.

**Table 1:** Experimental groups and treatments.

Groups	Treatments
Group I	Control
Group II	25 mg/kg b.w quercetin
Group III	50 mg/kg b.w quercetin
Group IV	16 mg/kg b.w AFM_2_
Group V	25 mg/kg b.w quercetin + 16 mg/kg b.w AFM_2_
Group VI	50 mg/kg b.w quercetin + 16 mg/kg b.w AFM_2_

**Figure 1:**
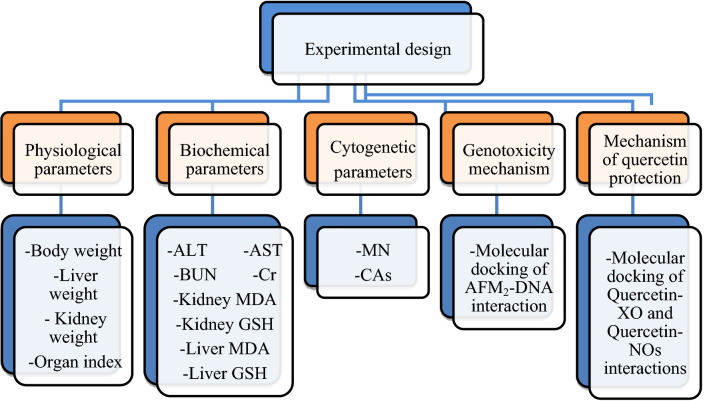
Experimental design. MDA: malondialdehyde, *GSH* Glutathione, *ALT* Alanine aminotransferase, *AST* Aspartate aminotransferase, *BUN* Blood urea nitrogen, *Cr* Creatinine, *MN* Micronucleus, *CAs* Chromosomal abnormalities, *XO* Xanthine oxidase, *NOs* Nitric oxide synthase.

Mice were brought to the laboratory environment where the experiments were to be carried out 7 days ago in order to adapt to environmental conditions. The water, feed, AFM_2_ and quercetin solutions given to each group were checked daily. At the end of the 28-day administration period, all mice were sacrificed. During the experiment, clinical symptoms of all animals such as activity, irritability, diarrhoea, wound formation and death were monitored daily. The effects of AFM_2_ and quercetin on albino mice were investigated with a multidisciplinary approach using different parameters, and the parameters used in the study are given in Fig. 1.

### Body weight, organ index, liver and kidney weights

Before the application period and at the end of the 28-day application period, each mouse was stunned under halothane anesthesia, body weights were measured and changes in body weight were determined based on the differences between before and after the application. At the end of the 28th day, the liver and kidney organs of the sacrificed mice were isolated and their weights were measured^12^. Eq. (1) was used to determine the organ index.

1 $${\text{Organ index}} = {\text{ organ weight}}/{\text{body weight x 1}}00$$

### Analysis of serum parameters

Since aflatoxins cause damage especially to the liver and kidney, which are the detoxification center, the markers of these two organs were examined. The effects of AFM_2_ toxicity and the protective role of quercetin in albino mice on serum parameters were determined by examining ALT, AST, BUN and creatinine levels in each group. For this purpose, whole blood samples from mice stunned under halothane anesthesia were collected in vacuum tubes and centrifuged at 1200 g at + 4 °C for 10 min to separate serum as a clear supernatant. AST (Teco Diagnostics, CAT. NO: A559–150), ALT (Teco Diagnostics, CAT. NO: A524–150), BUN (Teco Diagnostics, CAT. NO: B549–150) and creatinine (Teco Diagnostics, CAT NO: B549–150) NO: C513–480) levels were measured with the Medispec 99 M autoanalyzer using a commercial kit^13^.

### Antioxidant/oxidant dynamic

To determine the effects of AFM_2_ and quercetin on antioxidant/oxidant balance, MDA and GSH levels were measured in kidney and liver. For this purpose, liver and kidney organs isolated from each mouse sacrificed under halothane anesthesia were washed with sterile solution. Liver and kidney organs were homogenized in cold 0.15 M KCl solution. Homogenates were centrifuged at 5.000 g at 4 °C for 1 h and MDA and GSH analyzes were performed in the resulting supernatant. MDA and GSH levels were measured according to the colorimetric method proposed by Çavuşoğlu et al.^14^.

### Genotoxic and cytotoxic effects

The cyto-genotoxic effects of AFM_2_ and the protective effect of quercetin against these effects were determined by MN and CAs analysis. MN test was performed on buccal mucosa epithelium, erythrocyte and leukocyte cells. CAs analysis was investigated in bone marrow cells.

To determine the frequency of MN formed in the buccal epithelial cells, the mouths of the mice stunned with halothane anesthesia were rinsed with distilled water, cell samples were collected from the right and left buccal epithelium, and the cells were spread on a sterile slide. After the cells were fixed in methanol:acetic acid solution, they were stained with Feulgen and Fast Green and examined under a microscope. For MN test on erythrocytes, blood samples (5 µL) from the tail veins of mice stunned with halothane anesthesia were mixed with 3% EDTA solution and spread on sterile slides. The preparations fixed in 70% ethanol for 2 min were left to dry for 24 h. At the end of the period, the slides were stained with 5% Giemsa and analyzed under a microscope. For the leukocyte MN test, blood samples taken from each mouse were centrifuged at 5.000 g for 10 min, and 0.075 M KCl solution was added to the pellet. After incubation, the solution was centrifuged at 5.000 g for 10 min and a 3:1 methanol/acetic acid wash solution was added to the resulting pellet. After waiting at − 20 °C for 30 min, leukocyte cells were spread on sterile slides, stained with 5% Giemsa and examined under the microscope. 1.000 cells were analyzed in each group for MN frequency^13,15^.

The effects of AFM_2_ and quercetin on CA frequencies were investigated in the bone marrow cells. Mice treated with 0.025% colchicine were sacrificed 2 h later under halothane anesthesia. Bone marrow obtained from the femurs of mice was aspirated, washed with physiological solution and 0.075 M KCl was added. After fixation with Carnoy's solution, the samples stained with 5% Giemsa were examined under the microscope and CA frequencies were determined. 1.000 cells were analyzed for each group on the slides prepared for this purpose^16^.

### Molecular docking of AFM_2_-DNA

Potential interactions of AFM_2_ with different DNA sequences were investigated using molecular docking. DNA (PDB ID: 1cp8)^17^, B-DNA dodecamer (PDB ID: 1bna)^18^, and B-DNA dodecamer D (PDB ID: 195d)^19^ sequences were obtained from the protein database. The 3D structure of AFM_2_ (PubChem CID: 23,318) was obtained from PubChem. Energy minimization of the 3D structure of AFM_2_ was achieved with uff-force field using Open Babel v.2.4.0 software^20^. Insertion was performed using Autodock 4.2.6 software^21^ based on Lamarckian genetic algorithm. Placement analysis and 3D visualizations were made with Biovia Discovery Studio 2020 Client.

### Molecular docking of quercetin-XO and quercetin-NOs

The oxidative load-reducing effect of quercetin has an important place in its protective feature against oxidative damage. Quercetin achieves this effect by reducing the activity of enzymes that produce free radicals^22,23^. In this context, the interactions of quercetin with XO and NOs enzymes were investigated using molecular docking. The 3D structure of NOs (PDB ID: 1DWV)^24^ and XO (PDB ID: 3NRZ)^25^ were obtained from the protein data bank. The 3D structure of quercetin (PubChem CID: 5,280,343) was retrieved from the PubChem. It was prepared for molecular docking by determining the active sites of proteins, removing water molecules, and adding polar hydrogen atoms. For molecular docking processes in protein structures, chain A and HEM co-factor for nitric oxide synthase and chain C for xanthine oxidase were used in the process. Insertion was performed using Autodock 4.2.6 software^21^ based on Lamarckian genetic algorithm. Placement analysis and 3D visualizations were made with Biovia Discovery Studio 2020 Client.

### Protective effects of quercetin

The protective effects (PE) of quercetin against genotoxicity induced by AFM_2_ were calculated using Eq. (2). The investigated PE values depending on the dose were calculated separately for Group V and Group VI administered 25 mg/kg b.w and 50 mg/kg b.w quercetin.

2 $${\text{PE}}\left( \% \right) \, = \, \left[ {\left( {{\text{D}}_{{1}} - {\text{D}}_{{2}} } \right) \, /\left( {{\text{D}}_{{3}} - {\text{D}}_{{2}} } \right)} \right]{\text{ x 1}}00$$

D_1_: data of AFM_2_ + quercetin-treated group, D_2_: data of AFM_2_-treated group, D_3_: control data.

### Statistical analysis

Statistical analysis of the data obtained as a result of all analyzes was carried out using the SPSS for Windows V 22.0 (SPSS Inc, Chicago, IL, USA) program. One-way ANOVA and Duncan tests were used to evaluate the statistical differences between the experimental groups, respectively. Data were given as mean ± SD and were considered statistically significant at *p *< 0.05.

## Results and discussion

In this study, the toxic effects of AFM_2_ in albino mice and the protective role of quercetin against these effects were investigated with a multidisciplinary approach. There are many studies on aflatoxin toxicity in the literature, and these studies mainly focus on AFB_1_ toxicity. Information on AFM_2_ is very limited and studies are mostly related to residue analysis in milk and dairy products. AFM_2_ toxicity was determined by examining changes in body weight and organ weight, antioxidant/oxidant dynamics, serum parameters, which are markers of kidney and liver, and genotoxic effects. To elucidate the genotoxicity mechanism, the DNA-AFM_2_ interaction was investigated by molecular docking studies. The dose-dependent protective properties of quercetin against AFM_2_ toxicity and the mechanism of this effect were also investigated in the study.

### Body weight, organ index, liver and kidney weights

Body weight and organ weight are important indicators for determining the toxicities of xenobiotics and natural compounds. The effects of AFM_2_ and quercetin applications on organ index, body weight, liver and kidney weight in albino mice are given in Table 2. In the control group, an increase of 14.1 g in body weight was observed at the end of the 28-day administration period. Similarly, significant increases in body weight were also noted in Groups II and III, where only quercetin was administered. There was no statistically significant difference between these groups in terms of body weight gain (*p *> 0.05). Body weight decreased by 27.4% at the end of the 28th day in the AFM_2_ treated group compared to the control group (*p *< 0.05). There was no statistically significant difference in liver and kidney weights in the control group and only quercetin-administered groups in terms of both organ weights (*p *> 0.05). Liver weights of all three groups were measured in the range of 2.14–1.16 g, and kidney weights were measured in the range of 1.45–1.44 g. In the AFM_2_ applied group, liver and kidney weights decreased by 42.1% and 48.9%, respectively, compared to the control group, and these reductions were statistically significant (*p *< 0.05). Organ weight index, which expresses the ratio of organ weight to body weight, is also an important parameter used to evaluate the toxicity of any compound. Liver organ weight index decreased from 4.68 to 3.73 in the AFM_2_ group; kidney organ weight index decreased from 3.19 to 2.24. In many studies in the literature, it has been reported that reductions in body weight and organ index indicate the toxicity of test materials^26^. The adverse effects of AFM_2_ administration on body weight and organ weights in albino mice may be associated with decreased feed consumption, anorexia, disruptions in lipid anabolism and catabolism, and inhibitions in protein synthesis. In particular, impaired lipolysis-lipogenesis balance has an important place in changes in body weight, and disruptions in this balance cause abnormalities in weight gain^27^. Data on weight loss observed after exposure to aflatoxin in rabbits, mice and rats have also been reported in the literature. Adedara et al.^28^ reported 12% body weight loss in mice after aflatoxin exposure.

**Table 2:** Effect of AFM_2_ and quercetin on body, liver, kidney weights and organ index.

		Group I	Group II	Group III	Group IV	Group V	Group VI
B.W	IBW	31.96 ± 1.78	30.85 ± 1.64	31.59 ± 1.71	30.96 ± 1,66	31.48 ± 1.73	32.10 ± 1.81
	FBW	46.06 ± 2.32	44.81 ± 2.17	45.33 ± 2.24	33.46 ± 1.70	35.84 ± 1.85	39.30 ± 1.96
	TWG	14.10^a^	13.96^a^	13.74^a^	2.50^d^	4.36^c^	7.20^b^
O.W	LW	2.16 ± 0.45^a^	2.18 ± 0.42^a^	2.14 ± 0.43^a^	1.25 ± 0.28^d^	1.48 ± 0.35^c^	1.73 ± 0.39^b^
	KW	1.47 ± 0.42^a^	1.45 ± 0.40^a^	1.46 ± 0.43^a^	0.75 ± 0.24^d^	0.94 ± 0.30^c^	1.10 ± 0.36^b^
O.I	LOI	4.68	4.86	4.72	3.73	4.12	4.40
	KOI	3.19	3.23	3.22	2.24	2.62	2.79

Group I: control, Group II: 25 mg/kg b.w quercetin, Group III: 50 mg/kg b.w quercetin, Group IV: 16 mg/kg b.w AFM_2_, Group V: 16 mg/kg b.w AFM_2_ + 25 mg/kg b.w quercetin, Group VI: 16 mg/kg b.w AFM_2_ + 50 mg/kg b.w quercetin.

*BW* Body weight, *OW* Organ weight, *OI* Organ index, *IBW* Initial body weight, *FBW* Final body weight, *TWG* Total weight gain, *LW* Liver weight, *KW* Kidney weight, *LOI* Liver organ index, *KOI* Kidney organ index. Values are shown as mean ± SD (n = 6). Means shown with different letters^(a–d)^ on the same line are statistically significant (*p *< 0.05).

Quercetin administration exhibited an ameliorative effect on changes in body weight and organ index induced by AFM_2_ in mice. Quercetin administration together with AFM_2_ provided improvement in the regressions observed in body and organ weights. In the group administered 25 mg/kg b.w quercetin + AFM_2_, an increase of 7.1%, 18.4% and 25.3% were observed in body weight, liver and kidney organ weights, respectively, compared to the group administered only AFM_2_. In Group VI administered 50 mg/kg b.w quercetin + AFM_2_, these increases were determined as 17.4%, 38.4% and 46.6%, respectively. Quercetin has an effect on weight gain in living things by different mechanisms. Najafabadi et al.^29^ reported that quercetin caused improvements in weight gain in rats, and these improvements were associated with a wide variety of biological and therapeutic effects of quercetin. Azuma et al.^30^ reported that administration of quercetin in rats did not cause a change in body weight, but caused an increase in liver and kidney weights.

### Antioxidant/oxidant dynamic

**Figure 2:**
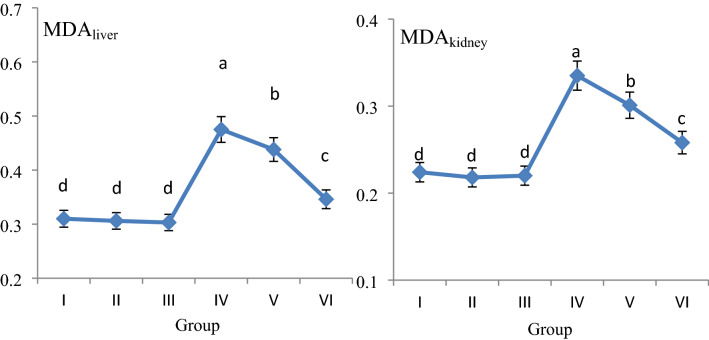
The effect of AFM_2_ and quercetin on MDA levels (nmol/g). Group I: control, Group I: control, Group II: 25 mg/kg b.w quercetin, Group III: 50 mg/kg b.w quercetin, Group IV: 16 mg/kg b.w AFM_2_, Group V: 16 mg/kg b.w AFM_2_ + 25 mg/kg b.w quercetin, Group VI: 16 mg/kg b.w AFM_2_ + 50 mg/kg b.w quercetin. Values are shown as mean ± SD (n = 6). Means shown with different letters^(a–d)^ are statistically significant (*p *< 0.05).

**Figure 3:**
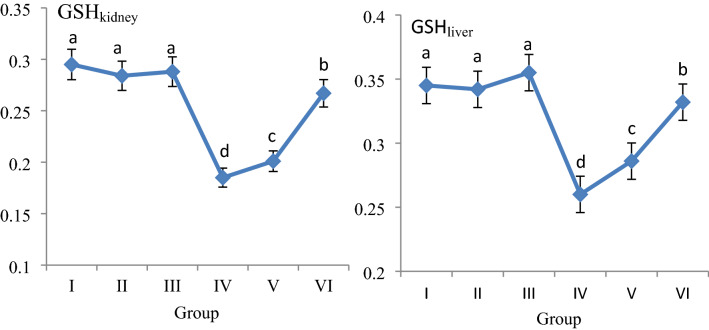
The effect of AFM_2_ and quercetin on GSH levels (mg/g). Group I: control, Group II: 25 mg/kg b.w quercetin, Group III: 50 mg/kg b.w quercetin, Group IV: 16 mg/kg b.w AFM_2_, Group V: 16 mg/kg b.w AFM_2_ + 25 mg/kg b.w quercetin, Group VI: 16 mg/kg b.w AFM_2_ + 50 mg/kg b.w quercetin. Values are shown as mean ± SD (n = 6). Means shown with different letters^(a–d)^ are statistically significant (*p *< 0.05).

The effects of AFM_2_ and quercetin administration on MDA and GSH levels in liver and kidney are given in Fig. 2 and Fig. 3, respectively. No significant changes were detected in the quercetin-alone treated groups compared to the control group, and MDA levels were in the range of 0.303–0.310 nmol/g; GSH levels were measured in the range of 0.342–0.355 mg/g in Groups I, II and III. No significant differences were found between these groups in terms of MDA and GSH levels (*p *> 0.05). While AFM_2_ administration increased liver MDA level by 53.2%, it caused 24.6% decrease in GSH level. In kidney, MDA level increased by 49.5% and GSH level decreased 37.2% after AFM_2_ application. These results indicate that the antioxidant/oxidant balance is impaired in the liver and kidney tissues. MDA is a mutagenic agent that is a product of lipid peroxidation. It occurs at very low levels in cells under normal conditions and is used in various biochemical pathways. High levels of MDA formation indicate the presence of oxidative stress and increased lipid peroxidation in the cell^31,32^. MDA, which increased significantly after AFM_2_ treatment compared to the control group, indicates that oxidative damage has occurred in the liver and kidney. It has been reported in the literature that aflatoxin applications increase lipid peroxidation in cells by inducing oxidative stress and causing cellular destruction^33^. Another evidence that AFM_2_ induces oxidative stress is the decreased levels of reduced GSH. GSH is an important endogenous antioxidant and has an important role in reducing oxidative stress in cells. As a strong antioxidant, GSH has an important role in the detoxification of various electrophilic compounds and peroxides through catalysis by GSH peroxidase and GSH transferase. GSH peroxidase detoxifies peroxides with GSH acting as an electron donor in the reduction reaction and GSSG is produced as the end product. The reduction of GSSG is catalyzed by GSH reductase in a process that requires NADPH. Keeping the oxidized or reduced GSH ratios (GSH: GSSG) in the cell at an optimal level is very important for the continuity of the cell. GSH deficiency makes the cell vulnerable to the risk of oxidative damage. It has been reported that GSH imbalance is observed in a wide variety of pathologies, including cancer, neurodegenerative disorders, cystic fibrosis, and aging^34^. Decreased GSH level and increased MDA level indicate that AFM_2_ induces oxidative damage in the liver and kidney, and as a result, the antioxidant/oxidant balance is impaired. Similarly, Shen et al.^33^ reported that AFB1 administration caused a significant, persistent and dose-dependent increase in MDA and conjugated diene levels, and significant decreases in GSH levels.

Quercetin + AFM_2_ treatment improved MDA and GSH levels compared to only AFM_2_-treated group and this improvement increased in a dose-dependent manner. AFM_2_ + 50 mg/kg b.w quercetin treatment caused 27.2% and 22.9% decrease in liver and kidney MDA levels, and 27.6% and 44.3% increase in GSH levels, respectively. These improvements in MDA and GSH levels also confirm the protective role of quercetin against oxidative damage. In the literature, there are studies that have determined the protective property of quercetin against damage induced by various chemicals. Yuan et al.^35^ observed that quercetin inhibited oxidative damage induced in mice and displayed a protective role by reducing stress-induced autophagy in the kidney. Pavanato et al.^36^ reported that 150 μmol/kg quercetin administration in rats provided protection against deterioration in liver histology, decreased collagen synthesis, increased lipid peroxidation and improved liver tissue integrity.

### Serum parameters

**Figure 4:**
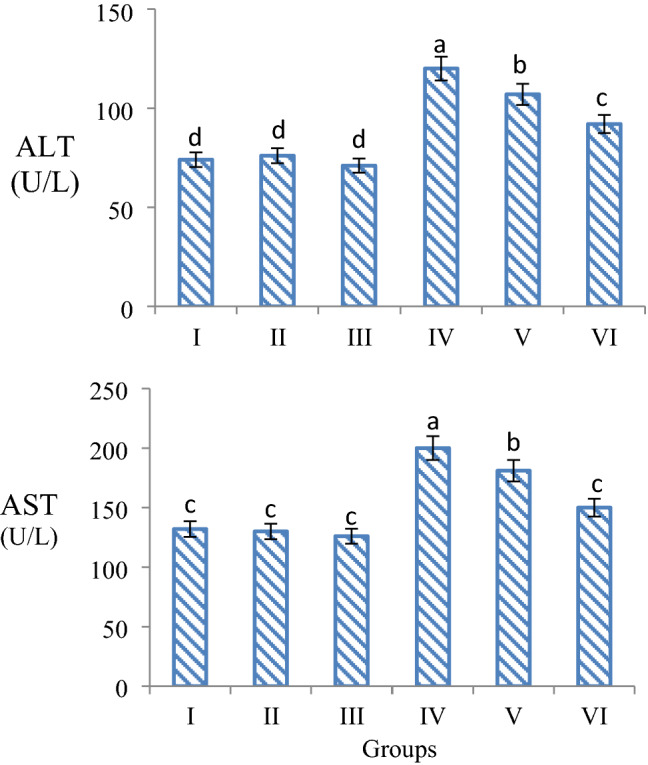
Effects of AFM_2_ and quercetin on serum ALT and AST levels. Group I: control, Group II: 25 mg/kg b.w quercetin, Group III: 50 mg/kg b.w quercetin, Group IV: 16 mg/kg b.w AFM_2_, Group V: 16 mg/kg b.w AFM_2_ + 25 mg/kg b.w quercetin, Group VI: 16 mg/kg b.w AFM_2_ + 50 mg/kg b.w quercetin. Means shown with different letters^(a–d)^ are statistically significant (*p *< 0.05).

ALT, AST, BUN and creatinine levels were investigated in serum samples obtained from mice as a result of experimental procedures. There was no statistically significant difference in serum parameters tested in only quercetin-administered groups and control group (*p *> 0.05). This result shows that quercetin administration alone does not cause any abnormality in serum parameters. It was determined that 16 mg/kg b.w AFM_2_ administration caused serious abnormalities in the tested serum parameters. AST and ALT levels increased by 34% and 23.3%, respectively, in the 16 mg/kg b.w AFM_2_-treated group compared to the control (Fig. 4). AST and ALT are important indicators of liver damage. Abnormal increases in the levels of these parameters indicate that AFM_2_ administration induces liver damage. The serum aminotransferases AST and ALT are intracellular enzymes that serve as markers of acute hepatocellular injury. AST is predominantly located in the mitochondria and cytosol of hepatocytes. AST is also found in heart and skeletal muscle, kidney, brain, pancreas, lung, leukocytes and erythrocytes. ALT is more specific to the liver compared to other tissues. AST and ALT are normally found at low levels in serum, and levels at higher concentrations indicate severe liver damage. Toxicity causing liver damage results in similar elevations of both AST and ALT^37,38^. The increase in both AST and ALT levels in the AFM_2_-treated group indicates liver damage in albino mice. It is known that aflatoxin species cause oxidative damage, hepatocellular necrosis, and many pathological and biochemical changes^39^. AST and ALT, which are intracellular enzymes, pass into the blood as a result of hepatocellular damage caused by AFM_2_ and their levels increase. Although studies on aflatoxin species are very diverse, there is no study investigating the effect of AFM_2_ on serum parameter levels in albino mice, but similar effects of other aflatoxin species are reported. Han et al.^40^ stated that AFB_1_, the most studied aflatoxin type, causes significant increases in serum ALT and AST levels. Mahmoud et al.^41^ found that there was a 47.6% increase in serum AST level and a 66.9% increase in ALT level in rats after exposure to aflatoxin.

**Figure 5:**
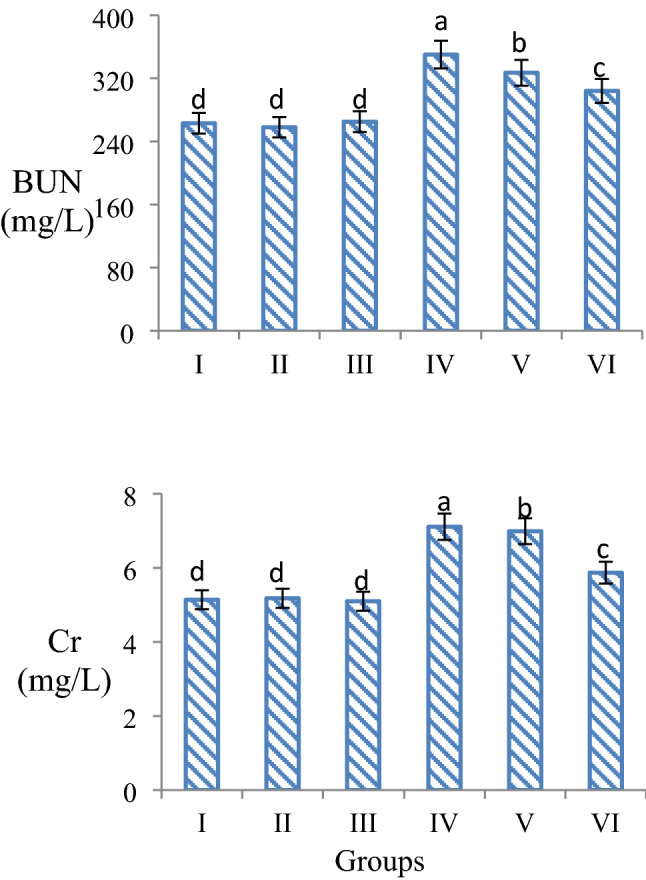
Effects of AFM_2_ and quercetin on serum BUN and creatinine levels. Group I: control, Group II: 25 mg/kg b.w quercetin, Group III: 50 mg/kg b.w quercetin, Group IV: 16 mg/kg b.w AFM_2_, Group V: 16 mg/kg b.w AFM_2_ + 25 mg/kg b.w quercetin, Group VI: 16 mg/kg b.w AFM_2_ + 50 mg/kg b.w quercetin. Means shown with different letters^(a–d)^ are statistically significant (*p *< 0.05).

Similar findings were found in serum parameters BUN and creatinine levels, which are indicators of kidney damage. While BUN and creatinine levels were at similar levels in the only quercetin-treated group and the control group, the BUN level increased by 24.8% and creatinine level by 27.7% in the 16 mg/kg AFM_2_-administered group compared to the control group (Fig. 5). Creatinine is a breakdown product of creatine phosphate in muscle and is produced by the body at a fairly constant rate, usually based on muscle mass. Creatinine is a commonly used indicator to measure kidney function. In chronic renal failure and uremia, there is a decrease in the removal of creatinine from the body by both the glomeruli and tubules, and the level of creatinine in the blood rises. Therefore, increases in serum creatinine levels indicate kidney failure and kidney damage. Serum creatinine values ​​are also affected by muscle function, muscle composition, activity, diet, and health status. Therefore, monitoring the BUN levels along with creatinine is very important in the evaluation of kidney functions. The amount of nitrogen in urea formed as a result of protein catabolism is expressed as BUN. BUN is a nitrogen-containing compound formed in the liver as the end product of protein metabolism and the urea cycle. BUN levels increase in cases of decreased renal clearance^42,43^. Aflatoxin exposure causes damage to glomerular capillaries, occlusion of cortical blood vessels, coagulation necrosis, inflammation, and focal bleeding in mammals. As a result of these effects, kidney damage and loss of function occur, and increases in serum BUN and creatinine levels are noted^44^. The increases observed in BUN and creatinine levels in the AFM_2_ applied group in this study indicate kidney damage. It is reported in the literature that aflatoxin administration increases BUN, creatinine and uric acid levels and induces kidney damage^45^. Mahmoud et al.^41^ found that rats fed bread containing aflatoxin had significant increases in serum urea and creatinine values, and they associated these increases with loss of renal tubular function.

Abnormal increases in ALT, AST, BUN and creatinine levels were observed to regress with quercetin administration. This regression demonstrates the protective ability of quercetin against AFM_2_ toxicity. The protective effect of quercetin is potentiated with dose, and the highest protection was observed in Group VI treated with 50 mg/kg b.w quercetin. AST, ALT, BUN and creatinine levels decreased by 13.6%, 24.3%, 15.5% and 14.2%, respectively, in the 50 mg/kg b.w quercetin + AFM_2_-treated compared to the group administered only AFM_2_. The increase in ALT and AST levels observed after AFM_2_ administration indicates liver damage, and the increase in BUN and creatinine levels indicates kidney damage. The suppression of the increase in these parameters by quercetin proves its protective effect on liver and kidney tissue. There is no study in the literature reporting the protective effect of quercetin against the change in serum parameters induced by AFM_2_, and this study is the first to report this property of quercetin. However, there are studies reporting the regulatory effect of quercetin against serum parameters that change as a result of induced oxidative damage. Chen^46^ determined that enzymes such as ALT, AST, ADH, γ-GT, which increased as a result of ethanol exposure in rats, decreased significantly after 5–20 mg/kg b.w quercetin administration, and that quercetin had a protective effect. Nabavi et al.^47^ reported that the increase in BUN and creatinine levels, which are signs of kidney damage in rats, decreased with the administration of 10 mg/kg b.w and 20 mg/kg b.w quercetin, and that quercetin provided a dose-dependent improvement.

### Cytogenetic effects

The cytogenetic effects of AFM_2_ and quercetin in different cells of albino mice were determined by investigating the frequencies of MN and CAs. The effects of AFM_2_ and quercetin on MN frequencies in different cells of albino mice are given in Table 3. While very low levels of MN formation were observed in the erythrocyte cells of the only quercetin-applied groups and control group of buccal epithelial cells, MN formations were not detected in the leukocyte cells of control and only quercetin-applied groups (*p *> 0.05). High rates of MN formation were detected in the buccal epithelium, erythrocyte and leukocyte cells after AFM_2_ exposure. The highest MN formation was observed in leukocyte cells treated with 16 mg/kg b.w AFM_2_ and MN were detected at a frequency of 65.72 ± 6.16. Among the three cells tested, the lowest level was found in buccal epithelial cells, with MN frequency of 25.36 ± 2.84. MNs originating from the remaining chromosomes in anaphase or chromosomal fragments in mitotic cells are an important indicator of toxicity. While increases in MN frequency indicate the presence of toxicity, MN size also provides information about the mechanism of toxicity. Small-sized MNs originate from acentric chromosomal fragments, while MNs originating from a lagging complete chromosome have larger sizes. Small MNs containing chromosomal fragments arise directly from DNA breaks, replication over a damaged DNA template, or inhibition of DNA synthesis (clastogenic effect). Large-sized MNs containing a complete chromosome arise as a result of damage to the spindle structure, kinetochore, centromere, or mitotic apparatus (aneugenic effect)^48^. The observation of small-sized MN formations in buccal epithelium and erythrocytes and large-sized MN formations in leukocyte cells after AFM_2_ application indicates that AFM_2_ has both clastogenic and aneugenic effects. In the literature, there are studies reporting that aflatoxins induce MN formations. Mahmoud et al.^41^ reported that MN formations were induced in bone marrow cells of rats fed bread containing aflatoxin, and aflatoxins significantly increased CAs and DNA fragmentation. One of the important findings of the MN test is that the application of quercetin together with AFM_2_ causes a regression in MN frequencies and this protective effect is dose-dependent. 50 mg/kg b.w quercetin provided 38.8%, 49.6% and 55.3% protection against MN formations in buccal epithelium, erythrocytes and leukocytes, respectively. This protective effect is closely related to the antioxidant properties of quercetin. Similarly, Tieppo et al.^49^ reported that 50 mg/kg b.w quercetin administration caused serious reductions in MN formation rates in rat bone marrow cells and provided significant protection.

**Table 3:** Effects of AFM_2_ and quercetin on MN frequencies in different cells of albino mice.

	Group	Buccal epithelium
	I	0.14 ± 0.02^d^
	II	0.00 ± 0.00^d^
	III	0.00 ± 0.00^d^
	IV	25.36 ± 2.84^a^
	V	18.24 ± 2.25^b^
	VI	15.50 ± 1.73^c^
	Group	Erythrocyte
	I	0.00 ± 0.00^d^
	II	0.13 ± 0.01^d^
	III	0.11 ± 0.01^d^
	IV	44.80 ± 4.18^a^
	V	30.85 ± 3.78^b^
	VI	22.56 ± 3.22^c^
	Group	Leukocyte
	I	0.00 ± 0.00^d^
	II	0.00 ± 0.00^d^
	III	0.00 ± 0.00^d^
	IV	65.72 ± 6.16^a^
	V	43.10 ± 5.73^b^
	VI	29.36 ± 4.85^c^

Group I: control, Group II: 25 mg/kg b.w quercetin, Group III: 50 mg/kg b.w quercetin, Group IV: 16 mg/kg b.w AFM_2_, Group V: 16 mg/kg b.w AFM_2_ + 25 mg/kg b.w quercetin, Group VI: 16 mg/kg b.w AFM_2_ + 50 mg/kg b.w quercetin. Normal appearance of buccal epithelium (a), buccal epithelium with MN (b), normal appearance of erythrocyte cell (c), erythrocyte cell with MN (d), normal appearance of leukocyte cell-lymphocyte (e), leukocyte with MN cell-lymphocyte (f). Means shown with different letters^(a–d)^ on the same line are statistically significant (*p *< 0.05).

AFM_2_ caused both MN formations and CAs as a result of aneugenic and clastogenic effects. The effects of AFM_2_ and quercetin applications on CAs frequencies detected in albino mice are given in Table 4. Statistically insignificant break, acentric chromosome and gap formation were observed in the control group and only quercetin-applied groups (*p *> 0.05). This result shows that 25 mg/kg b.w and 50 mg/kg b.w quercetin administrations did not induce CAs in bone marrow. High levels of break, fragment, gap, ring, acentric and dicentric chromosome formations were observed in the 16 mg/kg b.w AFM_2_ applied group. Among the CAs induced by AFM_2_, break and fragment were detected with a high frequency. These results indicate that AFM_2_ disrupts genome integrity and has a genotoxic effect. The frequencies of both MN and CAs increased in a dose-dependent manner. In short, dose increase increased genome instability, but this increase was not directly proportional. With the MN test performed in this study, it was determined that AFM_2_ has a clastogenic effect. The clastogenic effect causes strand breaks or chromosome breaks in DNA. Detection of the high rate of chromosomal breaks among CAs is closely related to the clastogenic effect. Breaks can be rearranged in the later stages of division and turn into other CAs. Chromosomal breaks may cause a high frequency of MN formations, ring chromosomes, acentric and dicentric chromosomes^50^. MN formations CAs observed in the AFM_2_ applied group confirm that the genotoxic effect was induced in the cell. This effect may occur as a result of the direct or indirect effects of AFM_2_. Especially with the induction of oxidative stress formation, base abnormalities, mismatches, disruption of helix structure and chain breaks that may occur in DNA can be considered as the source of CAs. However, AFM_2_ can also interact directly with DNA and produce a genotoxic effect. Many studies have reported in the literature that aflatoxin species cause chromosomal abnormalities, micronuclei formation, sister chromatid changes, chromosomal breaks and insertions^51,52^. Similarly, Fetaih et al.^53^ found that AFB_1_ exposure causes macro-DNA damages such as centromere rearrangements, breaks, deletions, gaps, sticky chromosomes, and hypopolyploidy.

**Table 4:** The effect of AFM_2_ and quercetin applications on CAs frequencies.

CA type	Group I	Group II	Group III	Group IV	Group V	Group VI
Break	0.19 ± 0.04^d^	0.00 ± 0.00^d^	0.00 ± 0.00^d^	45.19 ± 4.92^a^	31.87 ± 3.80^b^	24.15 ± 3.25^c^
Fragment	0.00 ± 0.00^d^	0.00 ± 0.00^d^	0.00 ± 0.00^d^	30.00 ± 3.90^a^	18.44 ± 2.96^b^	10.57 ± 2.33^c^
Acentric	0.00 ± 0.00^d^	0.00 ± 0.00^d^	0.11 ± 0.03^d^	21.20 ± 3.12^a^	15.38 ± 2.77^b^	8.52 ± 2.10^c^
Dicentric	0.00 ± 0.00^d^	0.00 ± 0.00^d^	0.00 ± 0.00^d^	12.96 ± 2.36^a^	7.78 ± 1.93^b^	3.36 ± 1.25^c^
Gap	0.25 ± 0.36^d^	0.19 ± 0.24^d^	0.00 ± 0.00^d^	9.24 ± 2.19^a^	5.17 ± 1.52^b^	3.21 ± 1.18^c^
Ring	0.00 ± 0.00^c^	0.00 ± 0.00^c^	0.00 ± 0.00^c^	6.30 ± 1.76^a^	3.86 ± 1.10^b^	3.14 ± 0.88^b^

Group I: control, Group II: 25 mg/kg b.w quercetin, Group III: 50 mg/kg b.w quercetin, Group IV: 16 mg/kg b.w AFM_2_, Group V: 16 mg/kg b.w AFM_2_ + 25 mg/kg b.w quercetin, Group VI: 16 mg/kg b.w AFM_2_ + 50 mg/kg b.w quercetin. Values are shown as mean ± SD (n = 6). For the frequency of CAs, 600 cells in each group were analyzed. Means shown with different letters^(a–d)^ on the same line are statistically significant (*p *< 0.05).

The decrease in the frequency of CAs observed in the groups administered quercetin together with AFM_2_ points to the protective role of quercetin against genotoxicity. With the increase in the dose of quercetin, increases in the protective properties against all chromosomal abnormalities were observed. The most prominent protective feature was observed in the administration of 50 mg/kg b.w quercetin, and protection against CAs types was obtained at different rates. In the literature, there are studies reporting the protective properties of quercetin against chromosomal damage. Horváthová et al.^54^ reported that quercetin administration provides 40–80% protection against CAs resulting from clastogenic effects in human melanoma cells.

### Molecular docking of AFM_2_-DNA sequences

**Figure 6:**
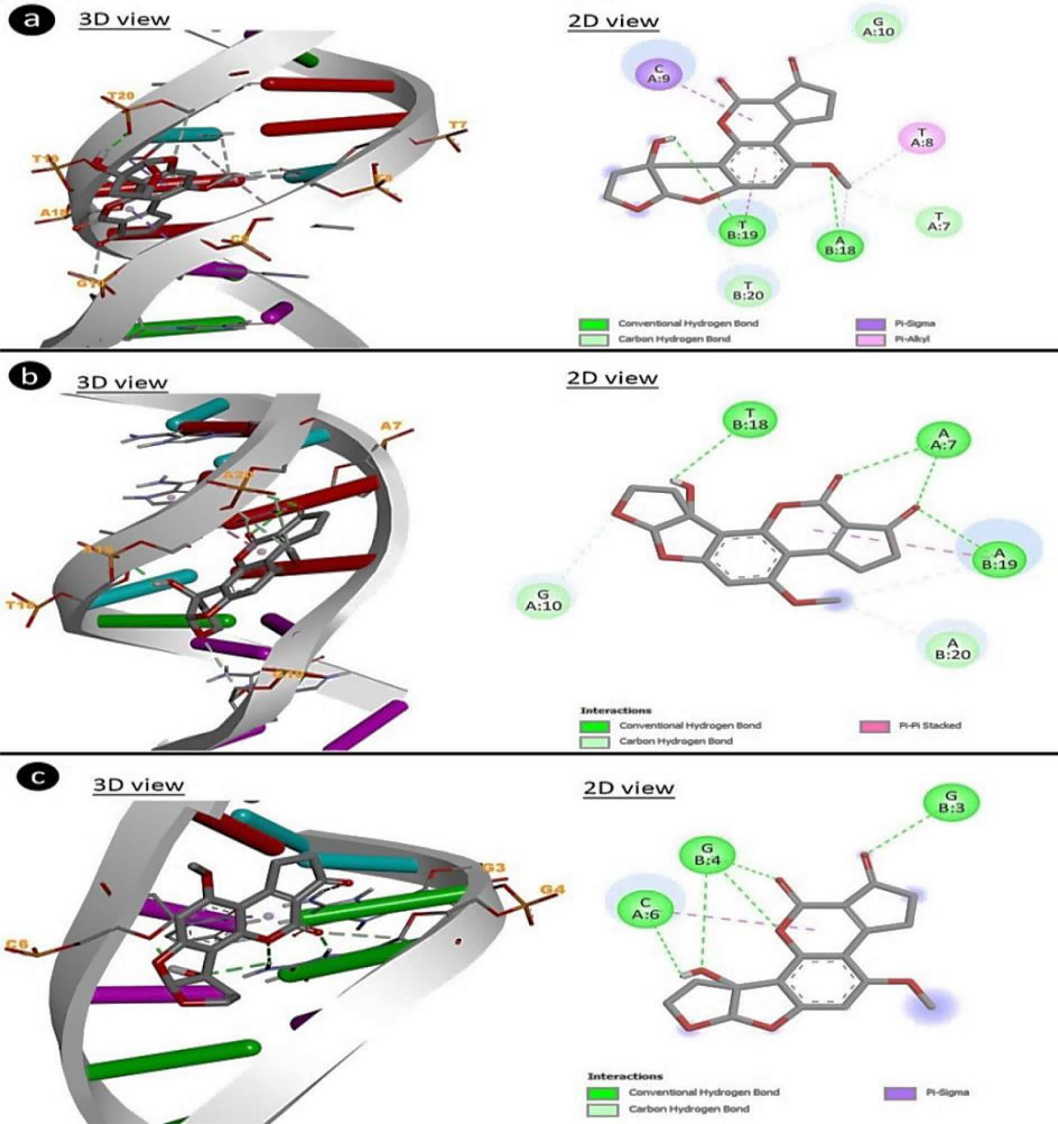
Molecular docking of AFM_2_ and DNA sequence. 1BNA (**a**), 195D (**b**), 1CP8(**c**).

To confirm the genotoxic effects of AFM_2_, its interactions with DNA molecules were analyzed by molecular docking and the results are given in Table 5 and Fig. 6. AFM_2_ showed molecular interactions with 1B-DNA by bases T7, T8 and C9 in the A chain and A18, T19 and T20 bases in the B chain. In this interaction, − 7.72 kcal/mol binding energy and 2.19 µM inhibition constant were calculated. AFM_2_ showed a binding energy of − 8.45 kcal/mol by interacting with B-DNA Dodecamer D by A7 and G10 bases in the A chain, and by T18, A19 and A20 bases in the B chain. In the interaction of AFM_2_ with DNA (1CP8), a binding energy of − 7.20 kcal/mol was formed and the interaction occurred with the C6 base in the A chain and the G3 and G4 bases in the B chain. According to the results of molecular docking analysis obtained in this study, AFM_2_ binds to DNA by interacting with regions rich in TTCG, ATT, TAA and GG nucleotides in DNA sequences and causes abnormalities in DNA structure. These interactions induce various CAs and MN formations during the division phases.

**Table 5:** Interaction constants of AFM_2_ and DNA sequences.

DNA	DNA sequence	Binding energy (kcal/mol)	Inhibition constant (Ki)	Interacting nucleic acid (chain: nucleotide)
B-DNA Dodecamer (1BNA)	5'-CGCGAATTCGCG-3'	− 7.72	2.19 uM	A:T7, A:T8 A:C9, A:G10 B:A18, B:T19,B:T20
B-DNA Dodecamer D (195D)	5'-CGCGTTAACGCG-3'	− 8.45	639.91 nM	A:A7, A:G10 B:T18, B:A19,B:A20
DNA (1CP8)	5'-TTGGCCAA-3'	− 7.20	5.24 uM	A:C6, B:G3 B:G4

### Protective effects of quercetin

**Figure 7:**
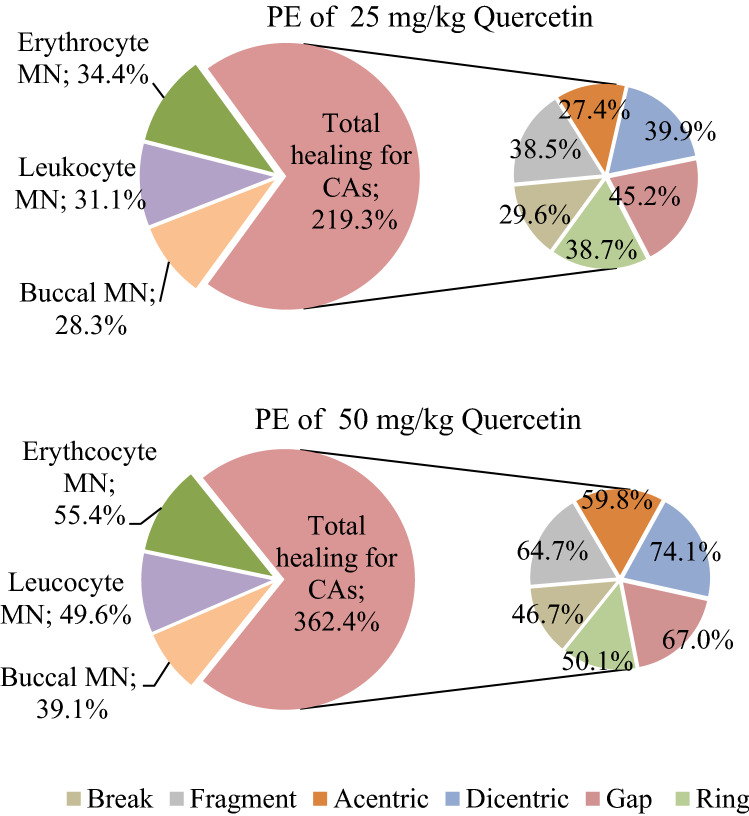
Protective effects (PE) of 25 mg/kg b.w and 50 mg/kg b.w quercetin applications against MN and CAs frequencies.

The dose-dependent protective effects of quercetin against AFM_2_ genotoxicity are given in Fig. 7. The protective effect of quercetin increased with the increase in the dose, but this increase was not directly proportional. While the highest protection was determined as 45.2% against gap formations in 25 mg/kg b.w quercetin application, 50 mg/kg b.w quercetin application provided 74.1% protection against dicentric chromosome formations. Considering all chromosomal abnormalities, 25 mg/kg quercetin provided protection in the range of 27.4–45.2%, while 50 mg/kg b.w quercetin provided protection in the range of 46.7–74%. Quercetin administration also showed significant protection against MN. While 25 mg/kg b.w quercetin caused 28.2%, 34.4% and 31.1% decreases in MN frequency of buccal epithelium, erythrocyte and leukocyte cells, respectively, these protective rates were 39.1%, 55.4% and 49.6% in 50 mg/kg b.w quercetin administration. The remedial role of quercetin against AFM_2_ toxicity is realized by different mechanisms. Quercetin acts as a powerful antioxidant substance by directly scavenging free radicals. This strong antioxidant property of quercetin originates from the catechol group in the molecule and especially from the -OH group in the 3rd position^55^. With the strong antioxidant effect of quercetin, oxidative stress induced by AFM_2_ is suppressed and cells are protected against damage. The fact that the increased MDA and decreased GSH levels in liver and kidney tissues observed in this study began to normalize with quercetin administration, indicating that quercetin suppresses oxidative stress. Similarly, Costa et al.^56^ reported that quercetin applications provide direct antioxidant protection by suppressing oxidative stress, and also induce cellular defense mechanisms against oxidative stress. Apart from this mechanism, quercetin also increases the antioxidant capacity of the body by regulating GSH levels. GSH is an important endogenous antioxidant compound that protects cells against oxidative damage. It has been reported that quercetin induces GSH synthesis in animal and cell studies^57^. AFM_2_ administration in liver and kidney tissues caused a significant decrease in GSH levels. In the groups administered quercetin together with AFM_2_, a dose-dependent improvement in GSH levels was observed in both kidneys and livers. This result shows that quercetin provides protection by regulating GSH levels. One of the important protective mechanisms of quercetin is the inhibition of free radical-forming enzymes in the cell. By inhibiting such enzymes with quercetin, the oxidative load in the cell is reduced. NOs and XO are two enzymes that produce free radicals in the cell, and these enzymes are inhibited by quercetin and the cell is protected against damage by reducing the oxidative load in the cell^23,58^. The interaction of quercetin-NOs and quercetin-XO were investigated in-silico by using molecular docking.

### Molecular docking of AFM_2_-XO and AFM_2_-NOs

**Figure 8:**
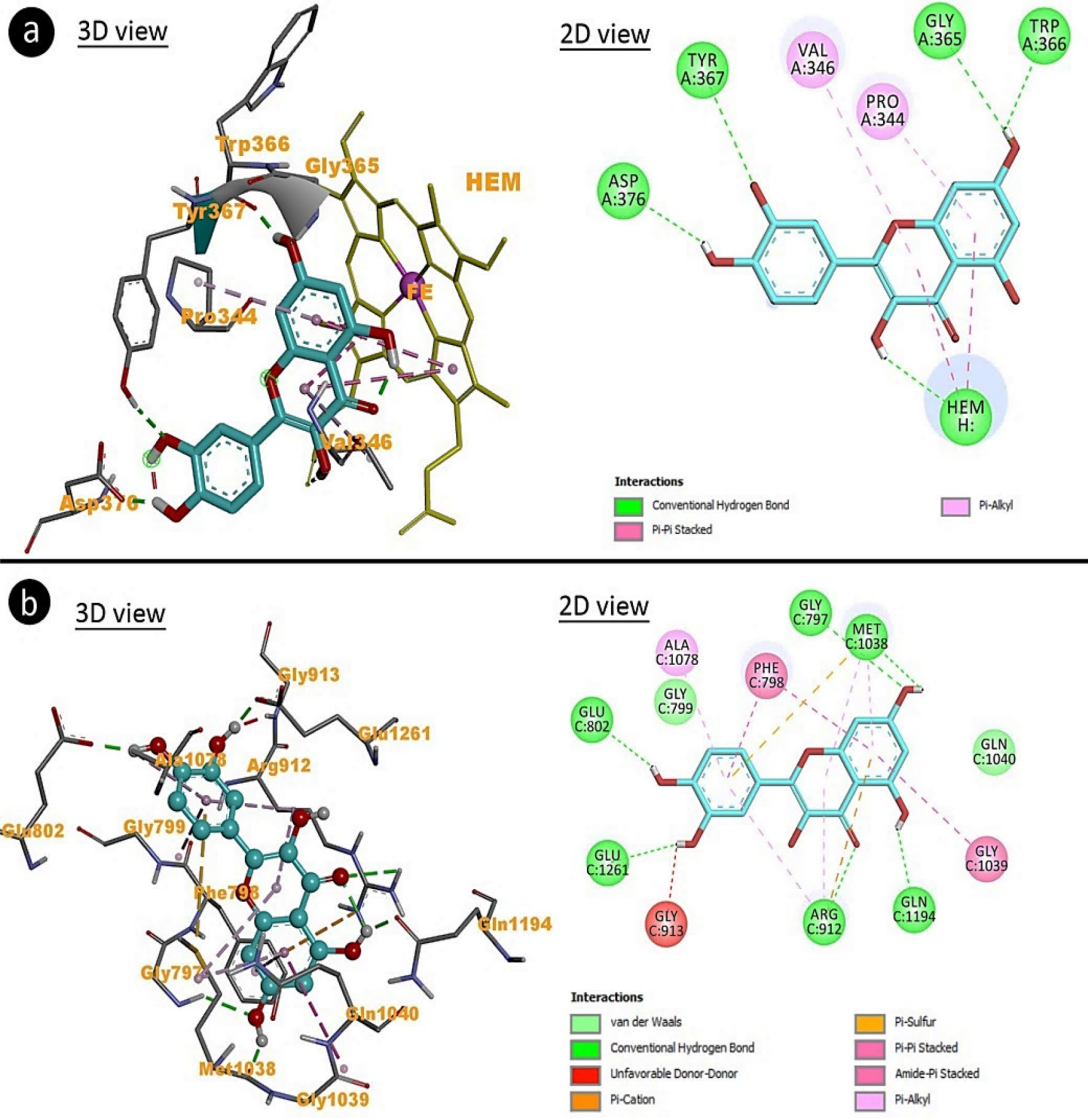
Molecular docking of AFM_2_-NOs (**a**) and AFM_2_-XO (**b**).

Quercetin reduces the oxidative load in the cell by causing inhibition of free radical-producing enzymes and exhibits antioxidant properties. It has been shown by molecular docking that quercetin, which has a protective role against AFM_2_ toxicity, interacts with NOs and XO enzymes (Table 6, Fig. 8). Quercetin interacts with the Val363 and Pro344 residues of NOs through hydrophobic interactions, forming hydrogen bonds over the Gly365, Tyr367, Asp376 and Trp366 residues. The binding energy of these interactions was calculated as − 8.93 kcal/mol and the inhibition constant as 283.71 nM. Similarly, the inhibition constant of these bindings of quercetin, which interacts with XO over different amino acids through hydrogen bonding and hydrophobic interactions, is 476.17 nM. Interactions between amino acids and quercetin in the polypeptide chain of enzymes cause changes in the three-dimensional structure and function of enzymes. The decrease in the activities of these enzymes reduces the formation of free radicals in the cell and protects the cell against oxidative damage.

**Table 6:** Interaction constants of AFM_2_ with NOs and XO enzymes.

Enzyme	Free binding energy (kcal/mol)	Inhibition constant (Ki)	Hydrogen bond interactions	Hydrophobic interactions
NOs	− 8.93	283.71 nM	GLY365, TYR367 ASP376, TRP366	VAL346, PRO344
XO	− 8.63	476.17 nM	GLY797, ARG912 GLU1261, GLU802 GLN1194, MET1038	PHE798(× 2), GLY799 GLN1040,GLY1039 ARG912,MET1038 (× 2) ARG912,ALA1078

Many studies in the literature have reported that quercetin prevents oxidative stress through the inhibition of free radical-producing intracellular enzymes^22,23^. Tiwari et al.^59^ investigated the interaction of quercetin with the polyketide synthase enzyme responsible for aflatoxin synthesis by molecular docking, and reported that enzyme inhibition as a result of the interaction could stop aflatoxin synthesis. Xu et al.^60^ reported that quercetin also has a strong inhibitory effect on other enzymes related to oxidative properties, such as acetylcholinesterase and butyrylcholinesterase.

**Figure 9:**
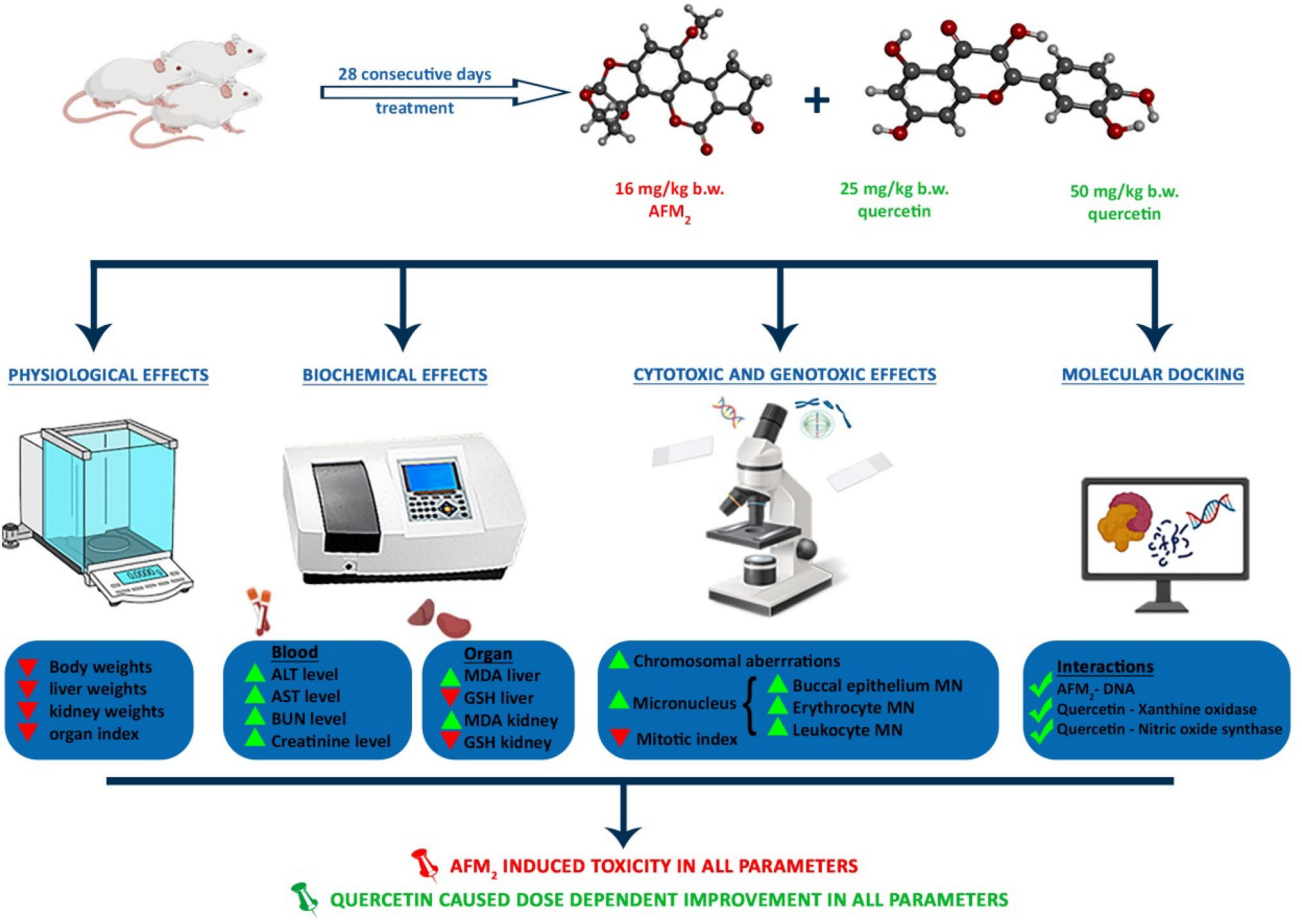
Experimental design and summary of findings.

All the parameters tested in the study and all the results obtained are summarized in Fig. 9.

## Conclusion

In this study, the potential toxic effects of AFM_2_, an important mycotoxin species, in albino mice and the protective role of quercetin against this toxicity were investigated. The fact that it has been detected in milk and dairy products frequently consumed by children in the developmental age makes it necessary to investigate the toxic effects of AFM_2_. AFM_2_, which caused serious changes in body weight, organ index, kidney and liver weight in albino mice, also caused the deterioration of antioxidant/oxidant balance in liver and kidney organs. The genotoxic effect of AFM_2_, which forms MNs and CAs in the bone marrow, has been associated with the DNA-AFM_2_ interaction elucidated by molecular docking. While the toxicity profile of AFM_2_ was revealed within the scope of the study, the protective feature of quercetin against this toxicity was also determined. Quercetin conferred significant protection by healing liver and kidney tissues of albino mice, providing partial normalization in serum parameter levels and drastic reductions in MN and CAs. These protective effects of quercetin are due to its antioxidant properties. Free radical-producing enzymes such as XO and NO increase the oxidative load in the cell. It was determined by molecular docking that quercetin interacts with these enzymes and causes inhibition. In this way, it reduced the oxidative load in the cell and provided antioxidant protection.

These results show the necessity of taking strategies and measures to prevent the consumption and contamination of milk and milk-based products contaminated with AFM_2_. In addition, awareness raising studies should be conducted on the effects of antioxidant supplements such as quercetin, which will reduce toxicity.

## Data Availability

The datasets used and/or analyzed during the current study are available from the corresponding author on reasonable request.
